# Data on internal cDNA amplification and color changes of the proteins derived from Pacific white leg shrimp shell

**DOI:** 10.1016/j.dib.2017.11.025

**Published:** 2017-11-10

**Authors:** Pan Chuang, Ishizaki Shoichiro, Nagashima Yuji, Gao Jialong, Watabe Shugo

**Affiliations:** a Graduate School of Marine Science and Technology, Tokyo University of Marine Science and Technology, 4-5-7 Konan, Minato, Tokyo 108-8477, Japan; b College of Food Science and Technology, Guangdong Ocean University, Haida Road 1, Mazhang, Zhanjiang 524088, Guangdong, China; c School of Marine Biosciences, Kitasato University, Sagamihara 252-0373, Japan

## Abstract

In this article, we report original data on the designation of the primers for full-length cDNA amplification and the internal cDNA amplification of red color-related pigment-binding protein derived from shrimp shell. Data on the color shifts of different soluble proteins under 100 °C 10 min heat treatment and the effects of heating temperatures (from 30 to 100 °C) on the color changes of crude water-soluble proteins are also included in this report. For further details and experimental findings please refer to the article “Isolation and cDNA cloning of a novel red color-related pigment-binding protein derived from the shell of shrimp, *Litopenaeus vannamei*” (Chuang et al., 2017) [1].

**Specifications Table**

**Table:** 

Subject area	*Food science, biochemistry, molecular biology*
More specific subject area	*Food biochemistry*
Type of data	*Tables, figure*
How data was acquired	*Colorimeter (CLR-7100F, Shimadzu, Kyoto, Japan), Nucleotide sequence (ABI 3130 Genetic Analyzer, Applied Biosystems, Foster City, CA, USA), Alignment of nucleotide sequences (ClustalW program,*htttp://clustalw.ddbj.nig.ac.jp*)*
Data format	*Raw and analyzed data*
Experimental factors	*Protein isolation and temperature setting*
Experimental features	*The amplification and alignment of internal cDNA sequence and the color changes of different soluble proteins were performed*
Data source location	*Tokyo, Japan*
Data accessibility	*The data are available with this article*

**Value of the data**

• Primer data can be used for a further understanding on the amplification of red color-related pigment-binding protein from shrimp shell.
• Sequence alignment data provide information on the sequence differences and is able to be compared with data from other authors when profiling the hemocyanins.
• Color shift data are valuable for the researchers interested in crustacean shell color change and color-related proteins derived from crustacean shells.

## 1 Data

**Fig. 1:**
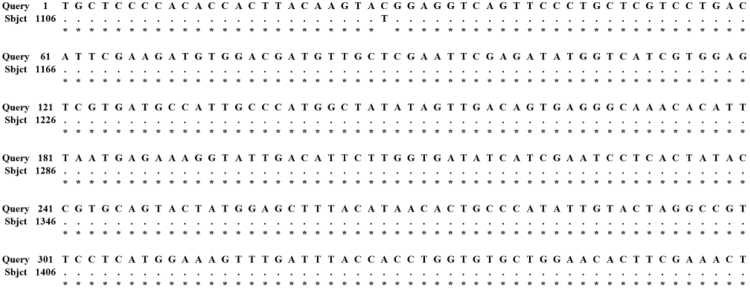
Data on the alignment of amplified internal fragment with known hemocyanin (KJ151291).

The data of this article provides information on the designation of primers used for different amplifications (Table 1) and the alignment of nucleotide sequences between red color-related protein derived from shrimp shell and known hemocyanin (KJ151291) (Fig. 1). Data on the color shifts of different soluble proteins under 100 °C 10 min heat treatment are presented in Table 2. Data on the effects of heating temperatures (30–100 °C) on crude water-soluble proteins are presented Table 3.

**Table 1:** Original experimental data on the nucleotide sequences of primers used in PCR amplification.

Primer^a^	Sequence (5′–3′)	Objective
Inter-F	TGCTCCCCACACCACTTACAAGTAC	Internal amplification
Inter-R	GTGGCAGTTTCRAAGTGTTCYAGCAC	
Gsp-R	GCAATGGCATCACGAATTCG	5′-end amplification
Gsp-F	TCCCAACGTGCAGTACTATG	3′-end amplification
5′-Gsp	GCACCATGAGGGTCTTAGTGGTTC	ORF amplification
3′-Gsp	TCACTAATGAATGTGTTCCCCATG	

a Meaning of letters: Inter, internal amplification primer; Gsp, gene-specific primer; F, forward primer; R, reverse primer.

**Table 2:** Original experimental data on the color shifts of different soluble proteins.

	Water-soluble		Salt-soluble		Acidic-soluble		Alkaline-soluble	
	Unheated	Heated	Unheated	Heated	Unheated	Heated	Unheated	Heated
L*	5.84±0.014^a^	5.17±0.014^b^	2.83±0.017^a^	1.82±0.024^b^	4.95±0.005^a^	5.03±0.045^a^	5.40±0.022^a^	5.59±0.033^b^
a*	−0.56±0.005^a^	0.01±0.005^b^	0.16±0.012^a^	0.14±0.029^a^	−0.29±0.012^a^	−0.30±0.019^a^	−0.19±0.012^a^	−0.21±0.012^a^
b*	0.76±0.016^a^	2.27±0.014^b^	1.74±0.021^a^	1.26±0.045^b^	0.92±0.025^a^	0.93±0.029^a^	2.82±0.029^a^	2.87±0.017^a^

Means followed by different lower-case letters within the same protein solutions differ significantly at *p*<0.05 versus unheated samples. Data are expressed as mean±standard deviation (*n*=3).

**Table 3:** Original experimental data on the color shifts of crude water-soluble proteins.

Temperature (°C)	L*	a*	b*
25 (control)	6.84±0.000^a^	−0.38±0.005^a^	1.15±0.005^a^
30	6.75±0.005^b^	−0.34±0.005^b^	1.11±0.005^b^
45	6.04±0.002^c^	−0.22±0.004^c^	1.46±0.009^c^
60	5.20±0.005^d^	0.05±0.002^d^	1.83±0.002^d^
80	3.79±0.005^e^	0.03±0.000^e^	2.09±0.005^e^
100	2.84±0.005^f^	0.33±0.000^f^	3.52±0.000^f^

Means followed by different lower-case letters within the same column differ significantly at *p*<0.05 versus the control. Data are expressed as mean±standard deviation (*n*=3).

## 2 Experimental design, materials and methods

Hemocyanin nucleotide sequences from *L. vannamei* (X82502), *P. monodon* (JF357966), *Fenneropenaeus chinensis* (FJ594414), *F. merguiensis* (KC920897), *Marsupenaeus japonicus* subunit L (EF375711) and subunit Y (EF375712) were aligned. Two internal primers (Inter-F and Inter-R) were designed based on the highly conserved zone corresponding to the amino acid regions of 270–278 and 381–389 (numbering is on the basis of the amino acid sequence of LvPBP75 shown in Fig. 3) [1]. The other gene–specific primers were all designed based on the determined nucleotide sequences.

Color shift of different soluble proteins was performed by heating at 100 °C for 10 min. Meanwhile, the red color change of crude-water soluble proteins were determined by heating at temperatures of 30, 45, 60, 80, and 100 °C for 10 min. Unheated samples were set as controls. Temperatures and color changes were monitored and recorded. Color changes were investigated by using the colorimeter (CLR-7100F, Shimadzu, Kyoto, Japan). The results are expressed as L* (brightness), a* (+a red, −a green), and b* (+b yellow, −b blue).

## References

[1] Chuang P., Shoichiro I., Yuji N., Jialong G., Shugo W. (2018). Isolation and cDNA cloning of a novel red color-related pigment–binding protein derived from the shell of shrimp, Litopenaeus vannamei. Food Chem..

