# Supplementary material for: Data on internal cDNA amplification and color changes of the proteins derived from Pacific white leg shrimp shell
Source: Data Brief. 2017 Nov 10;16:105–8. doi: 10.1016/j.dib.2017.11.025 (PMC5694961; doi:10.1016/j.dib.2017.11.025)
Supplement: Supplementary file 1 — Supplementary material [file mmc1.doc]

*Data article*

**Title:** *Data on internal cDNA amplification and color changes of the proteins derived from Pacific white leg shrimp shell*

**Authors:** Chuang Pan^a^, Shoichiro Ishizaki^a*^, Yuji Nagashima^a^, Jialong Gao^b^, Shugo Watabe^c^

**Affiliations:** ^a^ Graduate School of Marine Science and Technology, Tokyo University of Marine Science and Technology, 4–5–7 Konan, Minato, Tokyo 108–8477, Japan

^b^ College of Food Science and Technology, Guangdong Ocean University, Haida Road 1, Mazhang, Zhanjiang 524088, Guangdong, China

^c^ School of Marine Biosciences, Kitasato University, Sagamihara 252–0373, Japan

**Contact email:** Chuang Pan, silverpfoxc@hotmail.com

Yuji Nagashima, yujicd@kaiyodai.ac.jp

Jialong Gao, garonne–gjl@hotmail.com

Shugo Watabe, swatabe@kitasato-u.ac.jp

* Corresponding author:

Dr. Ishizaki Shoichiro

Graduate School of Marine Science and Technology, Tokyo University of Marine Science and Technology, Konan, Minato, Tokyo 108–8477, Japan

Tel./fax: +81–3–5463–0614

E–mail: ishizak@kaiyodai.ac.jp

**Abstract**

In this article, we report original data on the designation of the primers for full–length cDNA amplification and the internal cDNA amplification of red color–related pigment–binding protein derived from shrimp shell. Data on the color shifts of different soluble proteins under 100 ^o^C 10 min heat treatment and the effects of heating temperatures (from 30 to 100 ^o^C) on the color changes of crude water–soluble proteins are also included in this report. For further details and experimental findings please refer to the article “Isolation and cDNA cloning of a novel red color–related pigment–binding protein derived from the shell of shrimp, *Litopenaeus vannamei*” (P. Chuang, I. Shoichiro, N. Yuji, G. Jialong, W. Shugo, 2017) [1].

***Specifications Table***

| Subject area | *Food science, biochemistry, molecular biology* |
| --- | --- |
| More specific subject area | *Food biochemistry* |
| Type of data | *Tables, figure* |
| How data was acquired | *Colorimeter (CLR-7100F, Shimadzu, Kyoto, Japan), Nucleotide sequence (ABI 3130 Genetic Analyzer, Applied Biosystems, Foster City, CA, USA), Alignment of nucleotide sequences (ClustalW program, htttp://clustalw.ddbj.nig.ac.jp)* |
| Data format | *Raw and analyzed data* |
| Experimental factors | *Protein isolation and temperature setting* |
| Experimental features | *The amplification and alignment of internal cDNA sequence and the color changes of different soluble proteins were performed* |
| Data source location | *Tokyo, Japan* |
| Data accessibility | *The data are available with this article* |

**Value of the data**

- Primer data can be used for a further understanding on the amplification of red color–related pigment–binding protein from shrimp shell.
- Sequence alignment data provide information on the sequence differences and is able to be compared with data from other authors when profiling the hemocyanins.
- Color shift data are valuable for the researchers interested in crustacean shell color change and color–related proteins derived from crustacean shells.

**1. Data**

The data of this article provides information on the designation of primers used for different amplifications (Table 1) and the alignment of nucleotide sequences between red color–related protein derived from shrimp shell and known hemocyanin (**KJ151291**) (Fig. 1). Data on the color shifts of different soluble proteins under 100 ^o^C 10 min heat treatment are presented in Table 2. Data on the effects of heating temperatures (30–100 ^o^C) on crude water–soluble proteins are presented Table 3.

**Table 1**

Original experimental data on the nucleotide sequences of primers used in PCR amplification.

| Primer* | Sequence (5’–3’) | Objective |
| --- | --- | --- |
| Inter–F | TGCTCCCCACACCACTTACAAGTAC | Internal amplification |
| Inter–R | GTGGCAGTTTCRAAGTGTTCYAGCAC |
| Gsp–R | GCAATGGCATCACGAATTCG | 5’–end amplification |
| Gsp–F | TCCCAACGTGCAGTACTATG | 3’–end amplification |
| 5’–Gsp | GCACCATGAGGGTCTTAGTGGTTC | ORF amplification |
| 3’–Gsp | TCACTAATGAATGTGTTCCCCATG |

* Meaning of letters: Inter, internal amplification primer; Gsp, gene–specific primer; F, forward primer; R, reverse primer.

**Table 2**

Original experimental data on the color shifts of different soluble proteins

|  | Water–soluble | | Salt–soluble | | Acidic–soluble | | Alkaline–soluble | |
| --- | --- | --- | --- | --- | --- | --- | --- | --- |
|  | Unheated | Heated | Unheated | Heated | Unheated | Heated | Unheated | Heated |
| L* | 5.84 ± 0.014^a^ | 5.17 ± 0.014^b^ | 2.83 ± 0.017^a^ | 1.82 ± 0.024^b^ | 4.95 ± 0.005^a^ | 5.03 ± 0.045^a^ | 5.40 ± 0.022^a^ | 5.59 ± 0.033^b^ |
| a* | −0.56 ± 0.005^a^ | 0.01 ± 0.005^b^ | 0.16 ± 0.012^a^ | 0.14 ± 0.029^a^ | −0.29 ± 0.012^a^ | −0.30 ± 0.019^a^ | −0.19 ± 0.012^a^ | −0.21 ± 0.012^a^ |
| b* | 0.76 ± 0.016^a^ | 2.27 ± 0.014^b^ | 1.74 ± 0.021^a^ | 1.26 ± 0.045^b^ | 0.92 ± 0.025^a^ | 0.93 ± 0.029^a^ | 2.82 ± 0.029^a^ | 2.87 ± 0.017^a^ |

Means followed by different lower–case letters within the same protein solutions differ significantly at *p* < 0.05 versus unheated samples. Data are expressed as mean ± standard deviation (n = 3).

**Table 3**

Original experimental data on the color shifts of crude water–soluble proteins

| Temperature (°C) | L* | a* | b* |
| --- | --- | --- | --- |
| 25 (control) | 6.84 ± 0.000^a^ | −0.38 ± 0.005^a^ | 1.15 ± 0.005^a^ |
| 30 | 6.75 ± 0.005^b^ | −0.34 ± 0.005^b^ | 1.11 ± 0.005^b^ |
| 45 | 6.04 ± 0.002^c^ | −0.22 ± 0.004^c^ | 1.46 ± 0.009^c^ |
| 60 | 5.20 ± 0.005^d^ | 0.05 ± 0.002^d^ | 1.83 ± 0.002^d^ |
| 80 | 3.79 ± 0.005^e^ | 0.03 ± 0.000^e^ | 2.09 ± 0.005^e^ |
| 100 | 2.84 ± 0.005^f^ | 0.33 ± 0.000^f^ | 3.52 ± 0.000^f^ |

Means followed by different lower–case letters within the same column differ significantly at *p* < 0.05 versus the control. Data are expressed as mean ± standard deviation (n = 3).


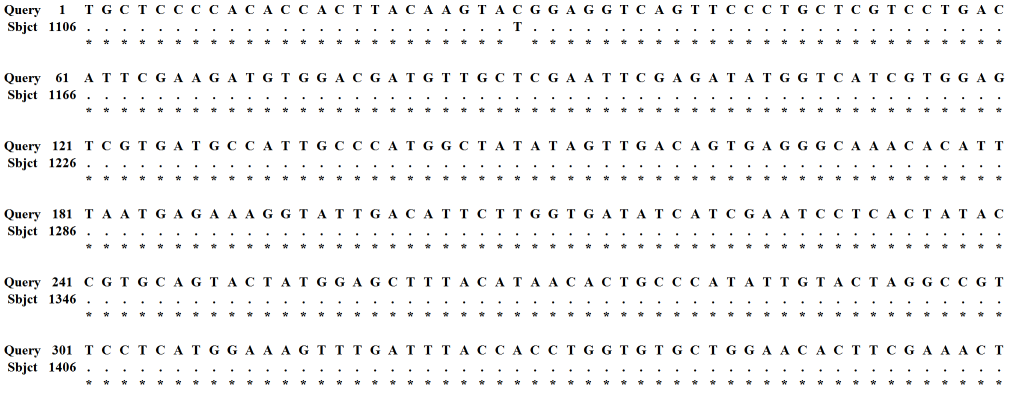


**Fig. 1.** Data on the alignment of amplified internal fragment with known hemocyanin (**KJ151291**).

1. **Experimental Design, Materials and Methods**

Hemocyanin nucleotide sequences from *L. vannamei* (**X82502**), *P. monodon* (**JF357966**), *Fenneropenaeus chinensis* (**FJ594414**), *F. merguiensis* (**KC920897**), *Marsupenaeus japonicus* subunit L (**EF375711**) and subunit Y (**EF375712**) were aligned. Two internal primers (Inter–F and Inter–R) were designed based on the highly conserved zone corresponding to the amino acid regions of 270–278 and 381–389 (numbering is on the basis of the amino acid sequence of LvPBP75 shown in Fig. 3) [1]. The other gene–specific primers were all designed based on the determined nucleotide sequences.

Color shift of different soluble proteins was performed by heating at 100 ^o^C for 10 min. Meanwhile, the red color change of crude–water soluble proteins were determined by heating at temperatures of 30, 45, 60, 80, and 100 °C for 10 min. Unheated samples were set as controls. Temperatures and color changes were monitored and recorded. Color changes were investigated by using the colorimeter (CLR−7100F, Shimadzu, Kyoto, Japan). The results are expressed as L* (brightness), a* (+a red, −a green), and b* (+b yellow, −b blue).

**Acknowledgements**

This work was partially funded by the Sasakawa Scientific Research Grant from The Japan Science Society (29–301).

**References**

[1] P. Chuang, I. Shoichiro, N. Yuji, G. Jialong, W. Shugo, Isolation and cDNA cloning of a novel red color–related pigment–binding protein derived from the shell of shrimp, *Litopenaeus vannamei*, Food Chem. 241(2018) 104–112.
